# Genome-wide comparative analysis of DNA methylation between soybean cytoplasmic male-sterile line NJCMS5A and its maintainer NJCMS5B

**DOI:** 10.1186/s12864-017-3962-5

**Published:** 2017-08-10

**Authors:** Yanwei Li, Xianlong Ding, Xuan Wang, Tingting He, Hao Zhang, Longshu Yang, Tanliu Wang, Linfeng Chen, Junyi Gai, Shouping Yang

**Affiliations:** Soybean Research Institute, National Center for Soybean Improvement, Key Laboratory of Biology and Genetic Improvement of Soybean (General, Ministry of Agriculture), State Key Laboratory of Crop Genetics and Germplasm Enhancement, Jiangsu Collaborative Innovation Center for Modern Crop Production, Nanjing Agricultural University, Nanjing, 210095 China

**Keywords:** Soybean (*Glycine max* (L.) Merr.), Cytoplasmic male sterility, DNA methylation, Differentially methylated gene, Gene expression

## Abstract

**Background:**

DNA methylation is an important epigenetic modification. It can regulate the expression of many key genes without changing the primary structure of the genomic DNA, and plays a vital role in the growth and development of the organism. The genome-wide DNA methylation profile of the cytoplasmic male sterile (CMS) line in soybean has not been reported so far.

**Results:**

In this study, genome-wide comparative analysis of DNA methylation between soybean CMS line NJCMS5A and its maintainer NJCMS5B was conducted by whole-genome bisulfite sequencing. The results showed 3527 differentially methylated regions (DMRs) and 485 differentially methylated genes (DMGs), including 353 high-credible methylated genes, 56 methylated genes coding unknown protein and 76 novel methylated genes with no known function were identified. Among them, 25 DMRs were further validated that the genome-wide DNA methylation data were reliable through bisulfite treatment, and 9 DMRs were confirmed the relationship between DNA methylation and gene expression by qRT-PCR. Finally, 8 key DMGs possibly associated with soybean CMS were identified.

**Conclusions:**

Genome-wide DNA methylation profile of the soybean CMS line NJCMS5A and its maintainer NJCMS5B was obtained for the first time. Several specific DMGs which participated in pollen and flower development were further identified to be probably associated with soybean CMS. This study will contribute to further understanding of the molecular mechanism behind soybean CMS.

**Electronic supplementary material:**

The online version of this article (doi:10.1186/s12864-017-3962-5) contains supplementary material, which is available to authorized users.

## Background

Cytoplasmic male sterility (CMS) is a common maternally inherited phenomenon that prevents the production of functional pollen [1]. In most cases of CMS, male-fertility can be recovered by specific gene named as restorer-of-fertility (RF) in the nuclear genome [2, 3]. At present, CMS has been present in more than 200 species of plants [4] and was widely used in crops hybrid breeding. Although the molecular mechanism of soybean CMS has been reported in transcriptomics [5], proteomics [6], microRNA [7] and mitochondrial genome [8] studies, the epigenetic regulation of the CMS remains poorly understood.

DNA methylation, a conserved epigenetic silencing mechanism in most eukaryote, could regulate the expression of many key genes, for example histone methylation along with gene silencing [9], RNA-directed DNA methylation repressing the LDMAR gene expression [10], and FLC chromatin with methylated modification delaying flowering [11]. In plant and animal, DNA methylation occurs predominately in CG enriched region of the genome, especially at CG island. However, cytosine methylation has also been observed in the CHG and CHH contexts with a low level in plant genome [12]. According to statistics, DNA methylation level varies from 4.6% to 30% in plant, which is relatively higher than that in animal [13]. No matter animal, plant or fungi, active genes are generally unmethylated, while transposable elements (TEs) are heavily methylated. So it was proposed that DNA methylation is roughly positively correlated with TE abundance, but negatively related to gene expression [14]. Within promoter region, DNA methylation is supposed to impact the transcriptional level of genes by silencing TE; whereas, within the gene body, DNA methylation may be associated with highly-expressed genes [15]. In addition, the establishment of DNA methylation mainly depends on DNA methyltransferases (DNMT), and DNA methylation regulation also relies on DNMT. To date, genes that encode DNMT have been isolated from a variety of plants, including rice, tobacco, corn and soybean [16–20].

Whole-genome sequencing and methylation profile has been widely reported in many plants, such as *Arabidopsis* [15], rice [21], soybean [14] and cotton [22]. Chen et al. [23] studied differentially methylation levels between the rice male sterile line PA64s and its fertile plant, and found the methylation level was higher in the sterile line than that of the fertile line. In addition, they also identified a differentially methylated gene (DMG) that was probably related to male sterility [24]. Liu et al. [25] proposed that the DNA methylation levels were higher in the corn CMS line than that in the maintainer line, and inferred that the changes of DNA methylation level in maize tassel may be associated with CMS. Although DNA methylome has also been used to analyze the distribution and average level of methylation in soybean root, stem, leaf and cotyledon [14], how DNA methylation may regulate the soybean CMS has no related report so far.

The soybean CMS line NJCMS5A was developed through consecutive backcross procedures with NJCMS1A [26–28] as the donor parent and Wandou 28 (designated as NJCMS5B afterwards) as recurrent parent in our laboratory. In this study, genome-wide comparative analysis of DNA methylation between soybean CMS line NJCMS5A and its maintainer NJCMS5B was conducted by whole-genome bisulfite sequencing. This is the first time to exploit epigenetic variation, and how gene expression is regulated in the whole genome of the soybean CMS.

## Results

### Analysis of genome-wide DNA methylation data of NJCMS5A and NJCMS5B

To study the genome-wide DNA methylation pattern of soybean, we collected flower buds from the soybean CMS line NJCMS5A and its maintainer NJCMS5B for constructing genomic DNA libraries. In total, 383,901,574 (NJCMS5A) and 398,207,546 (NJCMS5B) raw reads were generated from the two DNA library samples by whole-genome bisulfite sequencing (Table 1). After removal of related adapters, low-quality reads and containing Ns, 147,400,718 in NJCMS5A and 150,714,851 in NJCMS5B clean reads were collected (Table 1), of which 65.08% (NJCMS5A) and 62.10% (NJCMS5B) were uniquely mapped to the reference soybean genome of Williams82 (https://phytozome.jgi.doe.gov/pz/portal.html#!info?alias=Org_Gmax) (Table 1). Over 88% cytosines in soybean genome were covered by at least 5-fold coverage (Additional file 1). Meanwhile, more than 99% cytosines were converted, which indicates a high conversion rate in this study (Table 1).

**Table 1:** Summary of genome-wide methylation sequencing data

Sample	Raw reads	Clean reads	Total unique mapped reads	Percentage of mapped reads in total reads	The coverage of 5 × reads in total reads	Bisulfite sequencing conversion rate
NJCMS5A	383,901,574	147,400,718	95,930,507	65.08%	88.75%	99.75%
NJCMS5B	398,207,546	150,714,851	93,590,676	62.10%	88.48%	99.39%

**Fig. 1:**
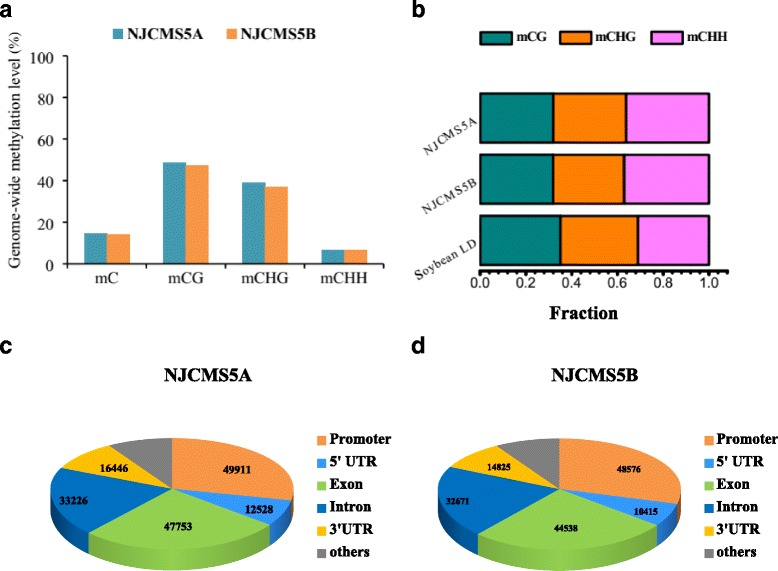
Feature of soybean genome-wide methylation between NJCMS5A and NJCMS5B. **a** Fraction of genome-wide methylated cytosines (mC) level and fraction of mC level in CG, CHG and CHH contexts. The blue column represented NJCMS5A, and red column represented NJCMS5B; **b** Distribution of methylated cytosines in the three contexts of NJCMS5A, NJCMS5B and soybean LD (soybean germplasm line LD00-2817P); **c** Number of methylated genes in the promoter, 5′UTR, exon, intron and 3′UTR region of NJCMS5A; **d** Number of methylated genes in the promoter, 5′UTR, exon, intron and 3′UTR region of NJCMS5B

The difference of DNA methylation level in the genome-wide between NJCMS5A and NJCMS5B was not significant. We detected 14.60% and 14.26% of methylated cytosines in NJCMS5A and NJCMS5B (Fig. 1a). Meanwhile, the average level of methylated cytosines in each context was also calculated for them. There were 48.58% of mCG (mCG/CG), 39.10% of mCHG (mCHG/CHG) and 6.74% of mCHH (mCHH/CHH) in NJCMS5A. Correspondingly, 47.37%, 37.27% and 6.73% of cytosines were methylated in CG, CHG and CHH contexts of NJCMS5B, respectively (Fig. 1a). Clearly, the average levels of mCG and mCHG were much higher than that of mCHH (Fig. 1a). And this distribution trend in three contexts in flower bud is generally similar to the trend reported previously in soybean root, steam, leaf and cotyledon [14]. Surprisingly, though the percent of mCHG was much lower than that of mCG and mCHG, the number of methylated cytosine sites in the three contexts was very similar. In NJCMS5A, we detected 48,495,434 mC sites, 15,497,738 mCG cites (32.0% of all mC), 15,425,507 mCHG sites (31.8% of all mC), 17,572,189 mCHH sites (36.2% of all mC), respectively; similarly, there were 31.9% mCG, 31.1% mCHG, and 37.0% mCHH in NJCMS5B (Fig. 1b). These results are consistent with the previous report about the DNA methylome of soybean LD00-2817P (“LD”) germplasm (Fig. 1b) [29]. So, we inferred there was a sizable proportion in of non-CG (CHG and CHH) in the plant cell.

### Profile of DNA methylation in gene and transposable element (TE) region

**Fig. 2:**
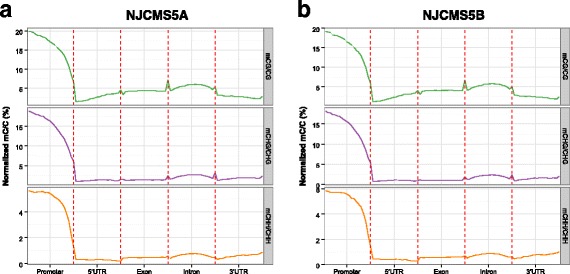
DNA methylation pattern in gene functional region under mC, mCG, mCHG and mCHH contexts. **a** Average density of DNA methylation in the promoter, 5′UTR, exon, intron and 3′UTR region under three contexts of NJCMS5A; **b** Average density of DNA methylation in the promoter, 5′UTR, exon, intron and 3′UTR region under three contexts of NJCMS5B. Each functional region was equally divided into 20 bins, and the mean of methylated cytosine density in each bin was defined as methylation density

The density distribution of methylated cytosines was tested to detect DNA methylation region. Firstly, from the chromosome level, DNA methylation was enriched mostly in the centromeric region, but with a little methylation was present at both ends of the chromosome (Additional file 2). Secondly, to better understand the relationship of DNA methylation and gene expression, we divided the genome into some functional regions, namely promoter region defined as the 2 kb region upstream of a transcription start site (TSS) and gene body consisting of 5′UTR, exon, intron and 3′UTR. For the promoter region, the DNA methylation level rapidly increased as departing from the TSSs sites in all contexts (Fig. 2). And it showed a low level in the gene body (Fig. 2). Besides this feature, when compared with CHH methylation level, CG and CHG methylation levels were significantly higher in promoter or gene body region (Fig. 2).

**Fig. 3:**
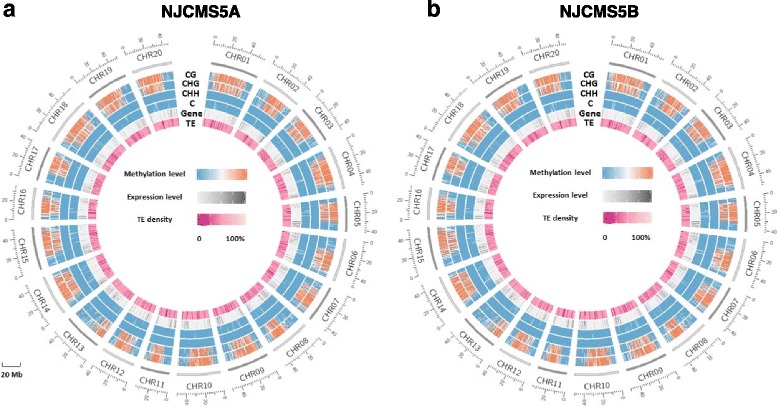
Circle plots of gene expression level, transposable element (TE) density and methylation level in the mC, mCG, mCHG and mCHH contexts of soybean (**a**) NJCMS5A and (**b**) NJCMS5B. TE indicated “TE density”; Gene indicated “gene expression level”; C, CG, CHH and CHG indicated “methylation level in mC, mCG, mCHG and mCHH, respectively”. Gene expression level was calculated by fragments per kilobase of exon model per million mapped fragments (RPKM). Data is plotted for 20 Mb windows in all soybean chromosomes (CHR01-CHR20). Gene expression level (from *light* colour to *dark* colour) indicates the level from 0 to 100%, and TE density (from *dark* colour to *light* colour) indicates the density from 0 to 100%

To further assess the correlations among DNA methylation, TE and gene expression, we collected genome sequences (from our laboratory) and TE sequences from SoyTEdb (http://www.soybase.org/soytedb/) [30, 31] (Fig. 3). On the whole, the pattern of DNA methylation in NJCMS5A was very similar to that in NJCMS5B (Figs. 2 and 3). The level of DNA methylation of the whole soybean genome was just about 14%. Except for C-G nucleotide pairs, most methylation occurred in CHG site (Figs. 1a and 3). In the CG, CHG and CHH contexts, DNA methylation was most abundant in TE-rich and gene-poor expression region in this study (Fig. 3). So DNA methylation may be positively related with TE density and negatively related to the expression level of genes in the soybean genome, which agrees with an idea proposed previously [29]. However, more empirical evidence is required to confirm that this hypothesis could be applied to any mC site detected in this study.

### Effect of DNA methylation on gene expression

Considered that the higher methylation level may reduce gene expression in the promoter region, we performed a further analysis of gene expression in different functional region using RNA-Seq data. Genes were divided into five groups, according to their expression level (Additional file 3): 0–1 (silent gene), 1–3 (low expressed gene), 3–15 (moderately expressed gene), 15–60 (highly expressed gene) and > 60 (gene with highest expression). The results clearly showed the negative correlation between DNA methylation and gene expression level in the promoter (Fig. 1c, d and Additional file 4). Due to large amounts of methylated genes (49,911 of NJCMS5A, 48,576 of NJCMS5B) occurred in promoter region, gene expression level in promoter was obviously lower than that in gene body (Fig. 1c, d and Additional file 4). However, in the gene body, the expression levels of high and middle genes were much high, which indicated no negative or positive correlation between gene expression and DNA methylation (Fig. 1c, d and Additional file 4).

### Identification of differentially methylated gene (DMG) between NJCMS5A and NJCMS5B

**Fig. 4:**
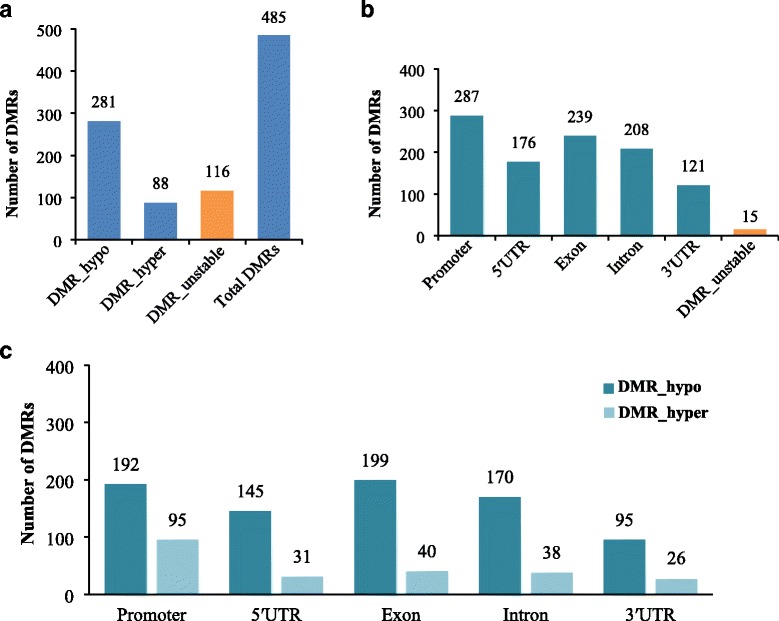
Distribution and proportion of differentially methylated genes (DMGs). **a** Total DMGs divided into hypo-DMGs and hyper-DMGs and unstable-DMGs (genes overlapping with both hyper- and hypo- DMGs); **b** Number of DMGs in gene functional region (promoter, 5′UTR, exon, intron and 3′UTR); **c** Number of hypo-DMGs and hyper-DMGs in gene functional region (promoter, 5′UTR, exon, intron and 3′UTR)

Differentially methylated region (DMR) is a significant sign of epigenetic variation, which may participate in regulating the DMGs to influence biological processes. By a genome-wide comparison of DNA methylation sequences between NJCMS5A and NJCMS5B, 3527 DMRs (Additional file 5) were identified by swDMR software (https://sourceforge.net/projects/swdmr/) with “FDR ≤ 0.05 and coverage changes ≥ 5” in this study. Then, DMGs were defined as genes overlapping with significant DMRs with at least 1 bp in the promoter and/or gene body. From 739 DMRs (Additional file 6) with genomic feature, we obtained 485 non-repeat DMGs including 281 hypo-methylated genes (57.9%) and 88 hyper-methylated genes (18.2%) in the NJCMS5A relative to NJCMS5B. In addition, 116 methylation-unstable genes (23.9%) that overlapped with different gene functional region (promoter, 5′UTR, exon, intron or 3′UTR) showed both hyper-methylation and hypo-methylation (Fig. 4a). In the gene functional region, there were more promoter-related DMGs (59.2%) than the exon-related (49.3%) or intron-related DMGs (42.9%) (Table 2 and Fig. 4b). In addition, we found the number of hypo-methylated DMGs was almost two to five times more than the number of hyper-methylated DMGs (Fig. 4c), which implied that DNA hypo-methylation often occurred as the epigenetic initial abnormality.

**Table 2:** Distribution of differentially methylated genes (DMGs) in gene functional region

Gene functional region	Promoter	5′ UTR	Exon	Intron	3′ UTR
DMG_hypo	192	145	199	170	95
DMG_hyper	95	31	40	38	26
Total DMG	287	176	239	208	121
DMG rate in genome (%)	59.2%	36.3%	49.3%	42.9%	24.9%

### Validation of the whole-genome bisulfite sequencing (WGBS) data by bisulfite treatment

**Fig. 5:**
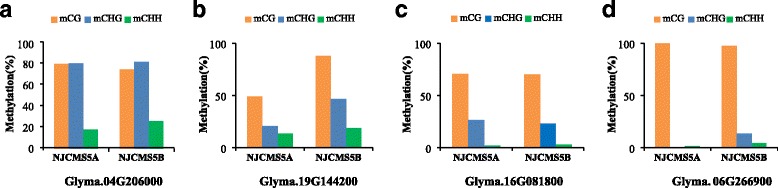
DNA methylation patterns of four differentially methylated genes (DMGs) validated by bisulfite treatment. **a** Glyma.04G206000 (oxysterol binding protein); **b** Glyma.19G144200 (basic helix-loop-helix DNA-binding protein); **c** Glyma.16G081800 (lipase); **d** Glyma.06G266900 (galacturonosyl transferase). The red, blue and green columns in the histograms refer to the collective methylation levels (in percentage) in CG, CHG and CHH contexts, respectively

**Fig. 6:**
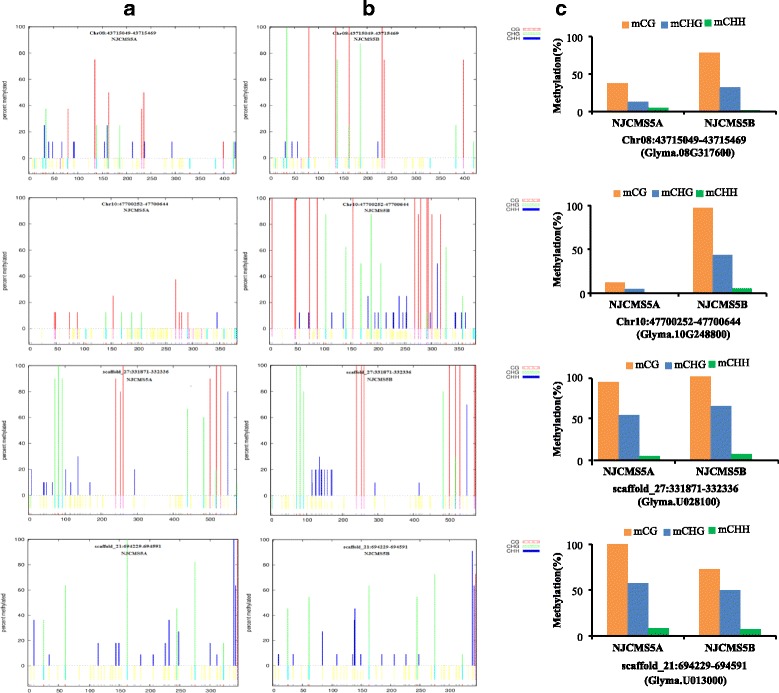
DNA methylation patterns of four typical differentially methylated regions (DMRs) with high methylation site. **a** Analysis of bisulfite treatment results of NJCMS5A; **b** Analysis of bisulfite treatment results of NJCMS5B. The colored bars above the x-axis show the methylated percent at each site. The short bars at the bottom of the graph show the position of the cytosines. The average level of methylation was calculated with 8–12 clones by bisulfite treatment and nested PCR. **c** Methylation patterns of DMRs, including hyper-methylated regions (Chr08:43,715,049–43,715,469, Chr10:47,700,252–47,700,644 and scaffold_27:331,871–332,336), and hypo-methylated region (scaffold_21:694,229–694,591)

We randomly selected 25 DMRs (16 hypo-DMRs and 9 hyper-DMRs) in NJCMS5A to confirm the WGBS data by bisulfite treatment, including *Glyma.04G206000* (OSBP), *Glyma.19G144200* (bHLH DNA-binding), *Glyma.16G081800* (lipase), *Glyma.06G266900* (galacturonosyl transferase) and so on (Figs. 5 and 6). Many of DMGs showed the cytosines in the CG and CHG contexts were more frequently methylated than that in the CHH context (Fig. 5 and Additional file 7). The bisulfite sequencing results showed that 21 DMRs containing of 13 hypo-DMRs and 8 hyper-DMRs were consistent with the WGBS data, which indicated WGBS data were credible in the study (Additional files 7 and 8). In term of CG context, many DMGs were methylated highly, so we also made models of the methylated percentage at each site (Fig. 6), including *Glyma.08G317600* (MYB domain protein), *Glyma.10G248800* (methyltransferases), *Glyma.U028100* (MYB domain protein) and *Glyma.U013000* (AGAMOUS). Among them, the gene of *Glyma.U013000* exhibited a higher methylation level in NJCMS5A compared NJCMS5B. But *Glyma.08G317600*, *Glyma.10G248800* and *Glyma.U028100* were identified to be hypo-methylation in NJCMS5A.

### Gene ontology (GO) annotation and Kyoto encyclopedia of genes and genomes (KEGG) pathway enrichment analysis

**Fig. 7:**
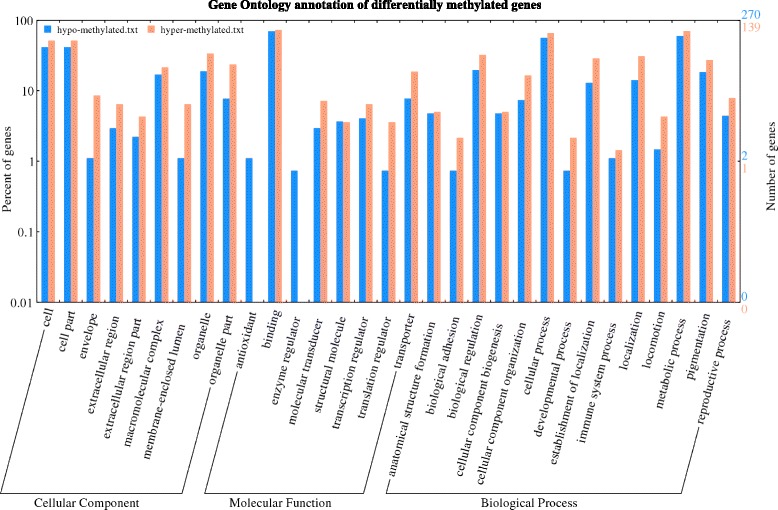
Gene Ontology (GO) annotation of differentially methylated genes (DMGs) between NJCMS5A and NJCMS5B. The *blue* and *red* column in the histogram referred to the hypo-methylation and hyper-methylation level (percentage and number), respectively

To describe property of genes and their products, WEGO (http://wego.genomics.org.cn) was used to functionally categorize the DMGs (Fig. 7). In total of 334 DMGs, comprising of 270 hypo-methylated and 139 hyper-methylated DMGs were annotated to 31 functional categories, including, 14 biological processes (BP), 9 cellular components (CC) and 8 molecular functions (MF) (Fig. 7). In the term of BP, we found that the hyper-methylated DMGs were mainly involved in catabolic process (GO: 0044238, GO: 0044237, GO: 0043170), cellular localization (GO: 0051641), macromolecule of localization (GO: 0033036) and transport (GO: 0006810) (Fig. 7). In the term of CC, the hyper-methylated DMGs were mostly participated in nucleoplasm part (GO: 0005654, GO: 0044451), sperm part (GO: 0036126, GO: 0097223, GO: 0097228) and organism membrane (GO: 0033644, GO: 0044218, GO: 0044279). And in the term of MF, the hyper-methylated DMGs were mostly involved in the process of transcription regulation (GO:0060089) and transporter (GO:0005215, GO:0022857) (Fig. 7). In addition, those hyper-methylation genes of NJCMS5A were made for GO enrichment analysis by using the GOseq R package. The result showed many of them appeared to be enriched in macromolecule modification, cellular metabolism, transcription regulation, protein phosphorylation and so on. These corresponding genes were taken into account for significantly down-regulated genes and further were listed (Table 3), including 86 genes for “BP” (36), 15 genes for “CC” (17) and 95 genes for “MF” (41).

**Table 3:** Significant Gene Ontology (GO) terms of hyper-methylated genes in NJCMS5A

GO accession	Term type	Description	DMR _ item	BG _ item	P _ value
GO:0019058	BP	viral infectious cycle	6	364	0.0022802
GO:0006011	BP	UDP-glucose metabolic process	1	1	0.0036546
GO:0015977	BP	carbon fixation	2	25	0.0037648
GO:0016042	BP	lipid catabolic process	3	109	0.0075620
GO:0044403	BP	symbiosis, encompassing mutualism through parasitism	8	789	0.0086707
GO:0071704	BP	organic substance metabolic process	76	16,860	0.0089794
GO:0044419	BP	interspecies interaction between organisms	8	796	0.0091147
GO:0006099	BP	tricarboxylic acid cycle	2	40	0.0094446
GO:0000723	BP	telomere maintenance	5	350	0.0094633
GO:0032200	BP	telomere organization	5	350	0.0094633
GO:0060249	BP	anatomical structure homeostasis	5	350	0.0094633
GO:0019067	BP	viral assembly, maturation, egress, and release	3	130	0.0121930
GO:0009060	BP	aerobic respiration	2	46	0.0123550
GO:0016032	BP	viral process	7	725	0.0175200
GO:0044238	BP	primary metabolic process	72	16,245	0.0190260
GO:0009225	BP	nucleotide-sugar metabolic process	1	6	0.0217300
GO:0034219	BP	carbohydrate transmembrane transport	1	6	0.0217300
GO:0071806	BP	protein transmembrane transport	2	66	0.0244210
GO:0006367	BP	transcription initiation from RNA polymerase II promoter	2	67	0.0251130
GO:0030150	BP	protein import into mitochondrial matrix	1	7	0.0253060
GO:0044764	BP	multi-organism cellular process	7	785	0.0256590
GO:0015074	BP	DNA integration	2	69	0.0265210
GO:0044237	BP	cellular metabolic process	67	15,179	0.0286360
GO:0072519	BP	parasitism	1	9	0.0324180
GO:0018149	BP	peptide cross-linking	1	10	0.0359550
GO:0006464	BP	cellular protein modification process	19	3357	0.0370220
GO:0036211	BP	protein modification process	19	3357	0.0370220
GO:0006468	BP	protein phosphorylation	15	2522	0.0427720
GO:0019068	BP	virion assembly	2	90	0.0430940
GO:0043170	BP	macromolecule metabolic process	57	12,853	0.0449750
GO:0043412	BP	macromolecule modification	20	3665	0.0451580
GO:0032940	BP	secretion by cell	3	221	0.0477090
GO:0046903	BP	secretion	3	221	0.0477090
GO:0006807	BP	nitrogen compound metabolic process	41	8794	0.0487850
GO:0000079	BP	regulation of cyclin-dependent protein serine/threonine kinase activity	1	14	0.0499750
GO:0071900	BP	regulation of protein serine/threonine kinase activity	1	14	0.0499750
GO:0016592	CC	mediator complex	4	183	0.0046636
GO:0036126	CC	sperm flagellum	1	2	0.0072960
GO:0097223	CC	sperm part	1	2	0.0072960
GO:0097228	CC	sperm principal piece	1	2	0.0072960
GO:0005654	CC	nucleoplasm	5	384	0.0136830
GO:0044451	CC	nucleoplasm part	5	384	0.0136830
GO:0071203	CC	WASH complex	2	61	0.0210820
GO:0033644	CC	host cell membrane	2	67	0.0251130
GO:0044218	CC	other organism cell membrane	2	67	0.0251130
GO:0044279	CC	other organism membrane	2	67	0.0251130
GO:0044441	CC	cilium part	1	7	0.0253060
GO:0036128	CC	catSper complex	1	9	0.0324180
GO:0030658	CC	transport vesicle membrane	2	87	0.0405380
GO:0019031	CC	viral envelope	4	354	0.0413760
GO:0036338	CC	viral membrane	4	354	0.0413760
GO:0005891	CC	voltage-gated calcium channel complex	1	13	0.0464890
GO:0034704	CC	calcium channel complex	1	13	0.0464890
GO:0004675	MF	transmembrane receptor protein serine/threonine kinase activity	2	18	0.0019525
GO:0008964	MF	phosphoenolpyruvate carboxylase activity	2	20	0.0024130
GO:0008234	MF	cysteine-type peptidase activity	5	273	0.0033820
GO:0019199	MF	transmembrane receptor protein kinase activity	2	25	0.0037648
GO:0001076	MF	RNA polymerase II transcription factor binding transcription factor activity	4	180	0.0043995
GO:0001104	MF	RNA polymerase II transcription cofactor activity	4	180	0.0043995
GO:0017111	MF	nucleoside-triphosphatase activity	17	2371	0.0059866
GO:0000988	MF	protein binding transcription factor activity	5	320	0.0065718
GO:0004611	MF	phosphoenolpyruvate carboxykinase activity	2	34	0.0068906
GO:0016462	MF	pyrophosphatase activity	17	2410	0.0070057
GO:0004197	MF	cysteine-type endopeptidase activity	4	221	0.0089745
GO:0016818	MF	hydrolase activity, acting on acid anhydrides, in phosphorus-containing anhydrides	17	2498	0.0098272
GO:0000989	MF	transcription factor binding transcription factor activity	4	228	0.0099793
GO:0003712	MF	transcription cofactor activity	4	228	0.0099793
GO:0016787	MF	hydrolase activity	38	7372	0.0141010
GO:0016817	MF	hydrolase activity, acting on acid anhydrides	17	2626	0.0154860
GO:0005516	MF	calmodulin binding	2	56	0.0179520
GO:0000149	MF	SNARE binding	1	5	0.0181410
GO:0019905	MF	syntaxin binding	1	5	0.0181410
GO:1,901,363	MF	heterocyclic compound binding	59	12,823	0.0194820
GO:0097159	MF	organic cyclic compound binding	59	12,831	0.0197370
GO:0004386	MF	helicase activity	9	1116	0.0217290
GO:0004308	MF	exo-alpha-sialidase activity	1	6	0.0217300
GO:0004683	MF	calmodulin-dependent protein kinase activity	1	6	0.0217300
GO:0016997	MF	alpha-sialidase activity	1	6	0.0217300
GO:0034062	MF	RNA polymerase activity	5	448	0.0247360
GO:0070403	MF	NAD+ binding	1	7	0.0253060
GO:0016779	MF	nucleotidyltransferase activity	7	785	0.0256590
GO:0003676	MF	nucleic acid binding	33	6489	0.0271790
GO:0003852	MF	2-isopropylmalate synthase activity	1	8	0.0288680
GO:0008083	MF	growth factor activity	2	73	0.0294300
GO:0003899	MF	DNA-directed RNA polymerase activity	4	319	0.0299720
GO:0003677	MF	DNA binding	23	4224	0.0332160
GO:0016298	MF	lipase activity	3	197	0.0359010
GO:0004325	MF	ferrochelatase activity	1	10	0.0359550
GO:0038023	MF	signaling receptor activity	4	347	0.0389240
GO:0047750	MF	cholestenol delta-isomerase activity	1	11	0.0394790
GO:0003678	MF	DNA helicase activity	6	693	0.0423700
GO:0016863	MF	intramolecular oxidoreductase activity, transposing C = C bonds	1	12	0.0429910
GO:0046915	MF	transition metal ion transmembrane transporter activity	3	221	0.0477090
GO:0046789	MF	host cell surface receptor binding	1	14	0.0499750

*BP* biological process; *CC* cellular component; *MF* molecular functional; *DMR* differentially methylated region; *BG*: background

Among the DMGs, 51 DMGs (Additional file 9) were predicted to be enriched in 35 biochemical metabolic pathways in the KEGG database (http://www.genome.jp/kegg/), including plant hormone signal transduction (ko04075), glycolysis / gluconeogenesis (ko00010), regulation of actin cytoskeleton (ko04810), RNA degradation (ko03018), cysteine and methionine metabolism (ko00270) and oxidative phosphorylation (ko00190). Based on the GO and KEGG pathway analysis, 8 genes were determined to be potentially related to soybean CMS (Table 4). For example, anther wall tapetum development gene *Glyma.U015500*, ATPase activity genes *Glyma.16G195100* and *Glyma.06G248800*, proteolysis gene *Glyma.U045200*, regulation of transcription genes *Glyma.08G317600*, *Glyma.U028100* and *Glyma.U040000* and mitochondrion structural gene *Glyma.14G212600*.

**Table 4:** Identified eight genes potentially related to cytoplasmic male sterility (CMS)

NO.	Gene ID	DMR region	Methylation status (NJCMS5A vs. NJCMS5B)	Description
1	Glyma.U015500	promoter	Hypo	bHLH DNA-binding super family protein
2	Glyma.16G195100	gene body	Hypo	mitochondrial mRNA modification
3	Glyma.06G248800	gene body	Hypo	ABC-2 type transporter family protein
4	Glyma.U045200	gene body promoter	Hypo	cysteine proteinases super family protein
5	Glyma.08G317600	promoter	Hypo	MYB domain protein 97
6	Glyma.U028100	promoter	Hypo	MYB domain protein 98
7	Glyma.U040000	promoter	Hyper	AP2/B3-like transcriptional factor family protein
8	Glyma.14G212600	gene body promoter	Hyper	PPR super family protein

*Hyper* DNA methylation level in NJCMS5A was higher than that in NJCMS5B; *Hypo* DNA methylation level in NJCMS5A was lower than that in NJCMS5B

### Validation of target DMGs by quantitative real-time PCR (qRT-PCR)

**Fig. 8:**
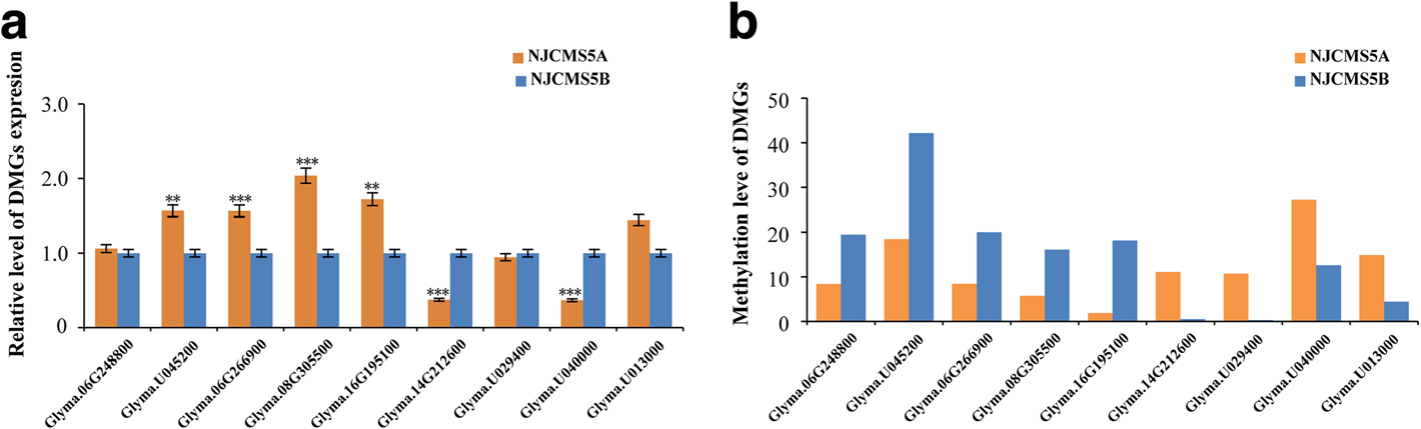
Expression level of nine DMGs validated by qRT-PCR. **a** qRT-PCR analysis of nine DMGs in NJCMS5A and NJCMS5B. X-axis represented gene name, the red column represented NJCMS5A, and blue column represented CK (NJCMS5B); Y-axis represented the relative level of gene expression. All qRT-PCR reactions were performed with three biological replicates; **b** DNA methylation level of nine DMGs in NJCMS5A and NJCMS5B

We also tried to conduct qRT-PCR to validate the relationship between DNA methylation and gene expression with 9 DMGs. As a result, 8 of 9 DMGs showed a negative correlation between DNA methylation and gene expression (Fig. 8). When the NJCMS5A methylation level of a DMG was significantly higher than that in NJCMS5B (*P* < 0.001) (Fig. 8b), the expression of the gene showed down-regulated in NJCMS5A, including *Glyma.14G212600* (PPR protein), *Glyma.U029400* (adenosine kinase) and *Glyma.U040000* (AP2/B3 transcriptional factor protein)*.* In addition, when the methylation level of a DMG in NJCMS5A was lower than that in NJCMS5B, the expression of the gene showed up-regulated in NJCMS5A, such as *Glyma.06G248800* (ABC transporter protein), *Glyma.U045200* (cysteine proteinases protein)*, Glyma.06G266900* (galacturonosyl transferase)*, Glyma.08G305500* (fimbrin-like protein) and *Glyma.16G195100* (mitochondrial mRNA modification). Only gene *Glyma.U013000* (AGAMOUS) had a high DNA methylation level and showed a high expression in NJCMS5A, which may be related to its function of inhibiting the down-stream gene expression, and it would be verified through the further research.

## Discussion

Although genome-wide DNA methylation map has been reported in many organism [15, 21, 22], DNA methylome of soybean CMS has few related studies. In this study, we apply DNA methylation sequencing on the soybean CMS line NJCMS5A and its maintainer NJCMS5B for the first time. And the methylation profile of flower bud was in line with the previously reported methylation profile of soybean root, steam, cotyledon and leaf [14]. The genomic DNA methylation data were obtained by whole-genome bisulfite sequencing from NJCMS5A and NJCMS5B (Table 1 and Fig. 1a), and it revealed that DNA methylation often occurred not only in CG context but also in non-CG (CHG and CHH) context throughout all chromosomes or genome functional region of soybean (Fig. 2), which is consistent with the DNA methylation level reported in other plants [14, 15, 23, 29].

DNA methylation is treated as an important epigenetic process that influences gene expression [9, 11]. In this study, the global methylation patterns of the two soybean lines are in agreement with previous observations [14, 29]. In addition, to further verify DNA methylation could silence gene expression in NJCMS5A, we select 9 DMRs to verify the negative correlation between DNA methylation and gene expression by qRT-PCR (Fig. 8). In addition, we identified 739 DMRs (Additional file 6) with genomic feature and 485 DMGs with 281 hypo-methylated genes (57.9%) and 88 hyper-methylated genes (18.2%) in the NJCMS5A relative to NJCMS5B (Fig. 4a). According to the gene region annotation, gene function, GO and KEGG pathway analysis, as well as previously reported studies of male sterility in plant, 8 key DMGs (Table 4) that may be related to soybean CMS were identified. And they were further discussed as follows.

### DMGs involved in regulation of pollen development potentially related to CMS

Numerous studies have shown that the pollen wall plays an important part in protecting pollen development [32]. The innermost layer of the anther wall (called tapetum) directly wrapped the microspore mother cell and microspore, and provided nutrition for microspore development by secreting large amounts of carbohydrate and lipid [33]. Therefore, tapetum played an important role in the process of pollen development, and the tapetum mutation may lead to pollen abortion [34–37]. The dysfunctional Tapertum1 gene (*DYT1*), encoding a basic helix-loop-helix (bHLH) TF, participated in tapetum development and protection [38]. In *Arabidopsis Dyt1 *mutant, because of the thicker callose wall, the pollen mother cell was unable to produce spores, leading to pollen abortion. In addition, the transcriptome analysis of *Arabidopsis* wild-type and *dyt1* mutant showed *dyt1* gene was in upstream of at least 22 coding bHLH TF genes, participating in regulating many specific metabolic pathways [39]. In this study, a DMG *Glyma.U015500* was found to encode a bHLH TF, which was a homolog of DYT1 in *Arabidopsis*. We supposed that *Glyma.U015500* may participate in the formation of pollen wall and may have an impact on pollen development in soybean.

The pollen coat is the outermost layer of the anther and it is mainly composed of sterol esters to protect the pollen [40]. The ATP binding cassette (ABC) transporter is in charge of transporting the coat materials for male gametophyte development [41]. In this study, a DMG *Glyma.06G248800* was supposed to encode an ABC-2 type transporter family protein, which was a homolog of ABCG9 of *Arabidopsis*. ABCG9 mainly participates in formation of the pollen coat with a high expression level in tapetum. However, mutations of *abcg9–1* and *abcg31–1* produced a lot of distorted and shrunken pollen grains, without mature pollen released [42]. So we considered *Glyma.06G248800* may contribute to the accumulation of sterol esters on the surface of the soybean pollen.

Together, abnormal methylation levels of *Glyma.U015500* and *Glyma.06G248800* in NJCMS5A may influence the development of the pollen wall, which may be a factor in the soybean CMS.

### DMGs involved in carbohydrate and energy metabolism potentially related to CMS

In the early stage of development, pollen is surrounded by tapetum. And in the late stage, tapetum begins to degrade [43]. Tapetum programmed cell death (PCD) plays a vital role in developing pollen. Cysteine Protease participates in plant PCD as the most common hydrolytic enzyme [44]. In this study, a DMG *Glyma.U045200* was predicted to encode a homolog of Cysteine Protease CEP1 of *Arabidopsis*. CEP1 participates in tapetum PCD and regulates the expression of FLOWERING LOCUS T (FT) in *Arabidopsis*. Excessive expression of CEP1 led to tapetum PCD degeneration in advance and pollen abortion [45], so proper CEP1 expression contributed to tapetum PCD for releasing the fertile pollen. *Glyma.U045200* showed a lower methylation level in NJCMS5A compared with NJCMS5B, which may promote the expression of gene *Glyma.U045200* in NJCMS5A, and eventually affected normal tapetum PCD, leading to pollen abortion.

### Transcription factor (TF) potentially related to CMS

In plant, TF plays a critical role in the regulation of plant metabolism and development by modifying the expression of their target genes [46]. For example, R2R3-type *MYB* gene could control many aspects of plant in secondary metabolism, as well as regulation of plant cell fate and identity [47]. *MYB98*, a member of the R2R3-MYB family TF, mainly regulates of transcriptional events in the synergid cell [48]. So it is necessary for pollen tube guidance and successful fertilization in flowering plant [49]. In the mature pollen of *Arabidopsis,* the expression of *MYB97* was much higher than normal, and a triple mutant of *myb97–1*, *myb101–2* and *myb120–3* caused overgrowth of the pollen tube into the embryo sac and disrupted sperm cell discharge, leading the failure of fertilization [50]. In this study, the DMG *Glyma.U028100* was predicted to encode a homolog of *MYB98*, and *Glyma.08G317600* was also predicted to encode a MYB TF protein. The promoter region of both DMGs showed a lower methylation levels in NJCMA5A compared with NJCMS5B, which implied that the abnormal expression of these two genes may disorder the development of floral organ, resulting in male sterility in NJCMS5A.

The MADS-box TF family is widespread in plant and animal, which involves in diverse and important biological functions [51], especially in floral organ process. Recently, soybean *GmMADS28* which encodes a MADS-box protein has been reported [52]. The genetically modified anther of 35S:*GmMADS28 *was not dehiscent and failed to release pollen, which largely led to plant male sterility [52]. In this study, the DMG *Glyma.U040000* was annotated as an AP2/B3-like TF family protein contained a B3 domain. In *Arabidopsis*, VRN1, as a member of the REM subfamily, contained two B3 DNA-binding domains. It could interact with AGAMOUS-like 20 (AGL20), flowering locus T (FT) and MADS-box protein flowering locus C (FLC), as well as with other regulators to restrain flowering [53]. The promoter region of *Glyma.U040000* with abnormal methylation level in NJCMS5A was supposed to negatively regulate gene expression, which may indirectly influence flower structure and flowering mechanism, leading to male sterility in NJCMS5A.

### DMGs encoding mitochondrial protein potentially related to CMS

Recently, it has also been proposed that the fertility of CMS plant could be restored by corresponding nuclear fertility restoration (RF) gene. And RF genes that were encoded by pentatricopeptide repeat (PPR) protein have been isolated from rice, carrot, and pepper [54–56]. RNA processing factor 1 gene (RPF1) belongs to a subgroup of PPR protein, which could guide RF gene product to restore CMS. In this study, the DMGs *Glyma.14G212600* and *Glyma.16G195100* were placed in the mitochondria. *Glyma.16G195100* was homologous with RPF1, which was supposed to play a role in fertility recovery in CMS plant [57]. In addition, the promoter of *Glyma.14G212600* was hyper-methylated in NJCMS5A compared with NJCMS5B, implying the expression of *Glyma.14G212600* may be suppressed. So, *Glyma.14G212600* and *Glyma.16G195100* were predicted to encode two PPR proteins, which may be associated with editing mitochondrial gene to impact the expression of the CMS-related genes in the mitochondria, resulting in CMS in NJCMS5A.

## Conclusion

In the study, the genome-wide methylation profiles of the soybean CMS line NJCMS5A and its maintainer NJCMS5B were obtained from whole-genome bisulfite sequencing. As a result, 739 DMRs and 485 DMGs, including 353 high-credible methylated genes, 56 methylated genes coding unknown protein and 76 novel methylated genes with no known function were identified. In addition, those valid methylated genes were identified as 281 hypo-methylated genes and 88 hyper-methylated genes in NJCMS5A relative to NJCMS5B. According to the gene region annotations, gene function, GO and KEGG pathway analysis, as well as the previous articles reported studies of male sterility in the plant, 8 key DMGs that may be related to soybean CMS were discussed, which mainly involved in participating in pollen development, encoding TF and the mitochondrial genome. This study provides DNA methylome of soybean CMS line and its maintainer line for the first time, which will contribute to further understanding the methylation mechanism in soybean CMS.

## Methods

### Plant materials

The soybean [*Glycine max* (L.) Merr.] CMS line NJCMS5A was developed through consecutive backcross procedures with NJCMS1A [26–28] as donor parent and Wandou28 (designated as NJCMS5B afterwards) as the recurrent parent. Both NJCMS5A and NJCMS5B were planted in the summer of 2014 at the Jiangpu Experimental Station, National Center for Soybean Improvement, Nanjing Agricultural University, Nanjing, Jiangsu, China. The male-sterile plant was identified through three kinds of methods including the dehiscence of anthers, germination rate of pollen, and performance of plant at maturity. Because it is very difficult to judge the precise development stage of pollen from the appearance of the flower buds in soybean, so during flowering period, the flower buds in different sizes were collected and pooled from NJCMS5A and NJCMS5B plant respectively, then immediately frozen in liquid nitrogen and stored at −80 °C for further use.

### Total DNA extraction and DNA library construction

Genomic DNA was extracted from the flower bud of NJCMS5A and NJCMS5B using DNeasy Plant Mini Kit (Qiagen Valencia, CA, USA), respectively, according to the manufacturer’s instruction. First, the purity of DNA sample was detected using the NanoPhotometer Spectrophotometer (Implen, CA, USA), and the concentration of DNA sample was measured using UV-Vis Spectrophotometer (Thermo Scientific, MA, USA). Subsequently, DNA sample was randomly sonicated to 200–300 bp using Covaris S220 (Thermo Scientific, MA, USA). After end repair and adenylation, the sonicated DNA fragments were ligated to cytosine-methylated barcodes according to manufacturer’s instruction. DNA fragment was treated twice with bisulfite using EZ DNA Methylation-Gold Kit (Zymo Research, CA, USA). After that, DNA library was constructed by PCR amplification. DNA library concentration was quantified by Qubit2.0 Fluorometer (Life Technologies, CA, USA), and the size of the insert fragment was tested by Biological Analyzer Agilent 2100 (Agilent, CA, USA). Finally, the qualified DNA library was sequenced on an Illumina Hiseq 2500 platform with paired-end reads by Novogene Bioinformatics Technology Co.Ltd. (Beijing, China).

### Bioinformatics analysis of DNA methylation sequencing data

After sequencing, the raw reads were filtered to remove adapters, Ns and low quality reads. The remaining reads called clean reads were stored in FASTQ format. Clean reads were aligned to the reference genome of soybean (https://phytozome.jgi.doe.gov/pz/portal.html#!info?alias=Org_Gmax) using Bismark software (version 0.12.5) with the default parameters [58]. The genetic structure annotation file was obtained from the public fit site of Phytozome (https://phytozome.jgi.doe.gov/pz/portal.html#!info?alias=Org_Gmax). Annotations of transposable element (TE) were downloaded from the SoyTEdb database (http://www.soybase.org/soytedb/) and TEs were plotted in 20 kb windows along the chromosome. Finally, only reads that were uniquely mapped to soybean reference genome were used for further analysis. mC percent (%) was calculated as the percentage of methylated C sites in the whole genome. mCG percent (%), mCHG percent (%) and mCHH percent (%) were calculated as the percentage of methylated C site in the C, CG and CHH contexts, respectively.

To identify the methylation site, we modeled the sum ***s*** ^**+**^_**i,j**_ of methylated counts as a binomial (Bin) random variable with methylation rate **r**_**i,j**_, ***s*** ^**+**^_**i,j ~**_ ***Bin*** **(** ***s*** ^**+**^_**i,j +**_ ***s*** ^**−**^_**i,j,**_ **,r**_**i,j**_ **)**. Then we employed a sliding-window approach, which is conceptually similar to the approaches used for bulk BS-Seq (http://www.bioconductor.org/packages/2.13/bioc/html/bsseq.html). With window size w = 3000 bp, step size = 600 bp [59] and sequencing depth ≥ 5, *q*-value < 0.05, the sum of methylated and unmethylated counts in every window were calculated. Else, the methylation level (ML) for each C site was defined as ***ML*** **(C) =** ***reads*** **(mC)/** ***reads*** **(mC + umC)**. The calculated ML was further corrected using with non-conversion rate r, ***ML***_**(corrected)**_ **= (** ***ML*** **–** ***r*** **)/(1 -** ***r*** **)** [60]. Density was calculated by ***sites*** **(mC)/** ***sites*** **(mC + umC)**. ML and density were both used for the analysis of the reads distribution in soybean chromosome and in the different genome functional region (promoter, 5′UTR, exon, intron, and 3′UTR) under three different contexts (CG, CHG and CHH).

Differentially methylated regions (DMRs) between NJCMS5A and NJCMS5B were identified using the swDMR software (https://sourceforge.net/projects/swdmr/) with a sliding-window approach [61]. The window is set to 1000 bp and step length is 100 bp. Fisher test is used to detect the DMRs with “FDR ≤ 0.05 and coverage changes ≥ 5”. Then the differentially methylated genes (DMGs) were collected from DMRs that overlapped gene functional region (promoter, 5′UTR, exon, intron and 3′UTR) with at least 1 bp. Next, Gene Ontology (GO) enrichment analysis (http://www.geneontology.org/) was performed for all the identified DMGs using the GOseq R package (*P*-value < 0.05) [62]. Finally, the hypergeometric test statistical method was used for the metabolic pathway analysis of all identified DMGs in Kyoto Encyclopedia of Genes and Genomes (KEGG) pathways (http://www.genome.jp/kegg/) using KOBAS software [63].

### Validation of the sequencing data by bisulfite treatment

Bisulfite genomic sequencing is regarded as a gold-standard for detection of DNA methylation, because it provides a qualitative and quantitative method to apply to a limit number of loci [64]. Twenty-five DMRs were selected randomly to validate the reliability of the sequencing data using a nest PCR (nPCR). All the primers (Additional file 10) were designed using the website of Kismeth (http://katahdin.mssm.edu/kismeth/revpage.pl), and synthesized commercially (Invitrogen, Shanghai, China). Briefly, 1 μg of genomic DNA was treated by bisulfite according to the protocol of the EZ DNA Methylation-Gold Kit (Zymo Research, CA, USA), and used as the template for the following nPCR amplification. Then, the bisulfite treatment PCR (BS-PCR) was performed in a 50 μl reaction mixture, containing 25 μl premix EX Taq DNA polymerase (TaKaRa, Osaka, Japan), 25 μg bisulfite-treated DNA and 0.2 μM of each pair of primers with a nest PCR. Next, products were purified using Gel Extraction Kit (Axygen, CA, USA), and cloned into the pMD19-T Simple Vector (TaKaRa, Osaka, Japan). Each DMR amplified with 10–14 clones was sequenced by Invitrogen Trading Shanghai Co.Ltd. (Shanghai, China), and the sequencing results were spliced and edited by BioEdit and vector NTI8 software, then analyzed in the Kismeth website.

### Validation of candidate DMGs by quantitative real-time PCR (qRT-PCR)

qRT-PCR was used to verify the methylated gene expression. All the primers (Additional file 11) were designed by Primer-BLAST, and synthesized commercially (Invitrogen, Shanghai, China). Firstly, 1 μg of total RNA was reverse-transcribed by reverse transcriptase according to the protocol of iScript cDNA Synthesis Kit (Bio-Rad, CA, USA), and used as the template for the following qRT-PCR amplification. Then, qRT-PCR was performed on the Bio-Rad CFX96 Touch q-PCR System (Bio-Rad, CA, USA) with iTaq Universal SYBR Green Super mix (Bio-Rad, CA, USA). Each reaction was replicated three times, and *β-*actin gene was used as the internal control gene. The relative level of gene expression was evaluated by the 2^–ΔΔCt^ method, NJCMS5B served as the control. The relative level of gene expression greater than 1 was regarded as up-regulated and less than 1 was regarded as down-regulated. Student’s *t*-test was adopted to test the difference between the relative level of gene expression of NJCMS5A and the control NJCMS5B. The means of 2^–ΔΔCt^ were considered significantly different at *P* < 0.05.

### Differential expression analysis of mRNA sequencing data

All the mRNA-seq analysis was based on the clean data with high quality. The expression quantity of each gene (fragments per kilobase of exon model per million mapped fragments, FPKM) was estimated by Cuffdiff software [65]. “FDR (False Discovery Rate) ≤ 0.05 [66, 67] and |Log2FC (Fold Change)| ≥ 1” were used as the threshold for judging the significant of gene expression difference.

## Additional files

Additional file 1: Data analysis of soybean genome-wide methylation sequencing. (PDF 207 kb)

Additional file 2: Density distribution of genome-wide methylation in soybean chromosome. (PDF 1510 kb)

Additional file 3: Gene expression level of transcriptome data. (XLS 8687 kb)

Additional file 4: Graph of gene expression level in gene functional region. (PDF 146 kb)

Additional file 5: Number of differentially methylated regions (DMRs) between NJCMS5A and NJCMS5B. (XLS 695 kb)

Additional file 6: Number of differentially methylated genes (DMGs) between NJCMS5A and NJCMS5B. (XLS 165 kb)

Additional file 7: Result of target DMRs in the CG, CHG and CHH context. (DOCX 22 kb)

Additional file 8: Validation of sequencing data by bisulfite treatment. (DOCX 16 kb)

Additional file 9: DMGs predicted to be enriched in KEGG pathways. (DOCX 18 kb)

Additional file 10: Primer pairs used for bisulfite treatment. (DOCX 21 kb)

Additional file 11: Primer pairs used for quantitative real-time PCR (qRT-PCR). (DOCX 17 kb)

## Abbreviations

bHLH: basic helix-loop-helix
BP: biological process
CC: cellular component
CMS: cytoplasmic male sterility
DMG: differentially methylated gene
DMR: differentially methylated region
DNMT: DNA methyltransferase
DYT1: dysfunctional tapetum 1
GO: Gene Ontology
KEGG: Kyoto Encyclopedia of Genes and Genomes
mC: methylated cytosine
MF: molecular function
PCD: programmed cell death
PPR: pentatricopeptide repeat-containing
qRT-PCR: quantitative real-time PCR
RF: fertility restoration
RPF1: RNA processing factor 1
TE: transposable element
TF: transcription factor
WGBS: whole-genome bisulfite sequencing
