# Supplementary material for: Genome-wide comparative analysis of DNA methylation between soybean cytoplasmic male-sterile line NJCMS5A and its maintainer NJCMS5B
Source: BMC Genomics. 2017 Aug 10;18:596. doi: 10.1186/s12864-017-3962-5 (PMC5557475; doi:10.1186/s12864-017-3962-5)
Supplement: Supplementary file 1 — Data analysis of soybean genome-wide methylation sequencing. (PDF 207 kb) [file 12864_2017_3962_MOESM1_ESM.pdf]

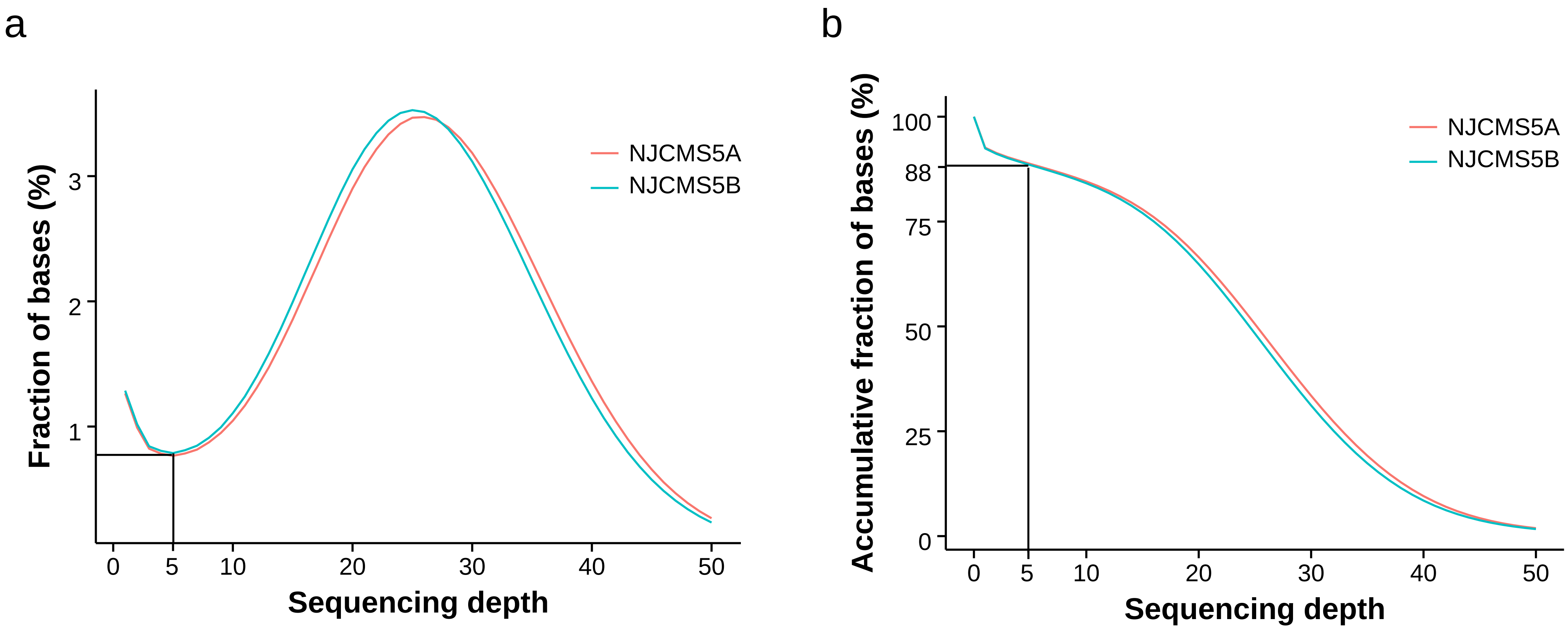

Figure S1 Data analysis of soybean genome-wide methylation sequencing. (a) Coverage of each signle-base site on the genome both NJCMS5A and NJCMS5B; The x-axis represents the coverage of sequencing depth; the y-axis represents the percentage of each signle-base site in the whole genome (Yi); **(b)** Accumulative coverage of genome sequencing both NJCMS5A and NJCMS5B; The y-axis represents the accumulative percentage of each signal-base site in the whole genome (Yj) Yj>=Yi.
