# Supplementary material for: Genome-wide comparative analysis of DNA methylation between soybean cytoplasmic male-sterile line NJCMS5A and its maintainer NJCMS5B
Source: BMC Genomics. 2017 Aug 10;18:596. doi: 10.1186/s12864-017-3962-5 (PMC5557475; doi:10.1186/s12864-017-3962-5)
Supplement: Supplementary file 2 — Density distribution of genome-wide methylation in soybean chromosome. (PDF 1510 kb) [file 12864_2017_3962_MOESM2_ESM.pdf]

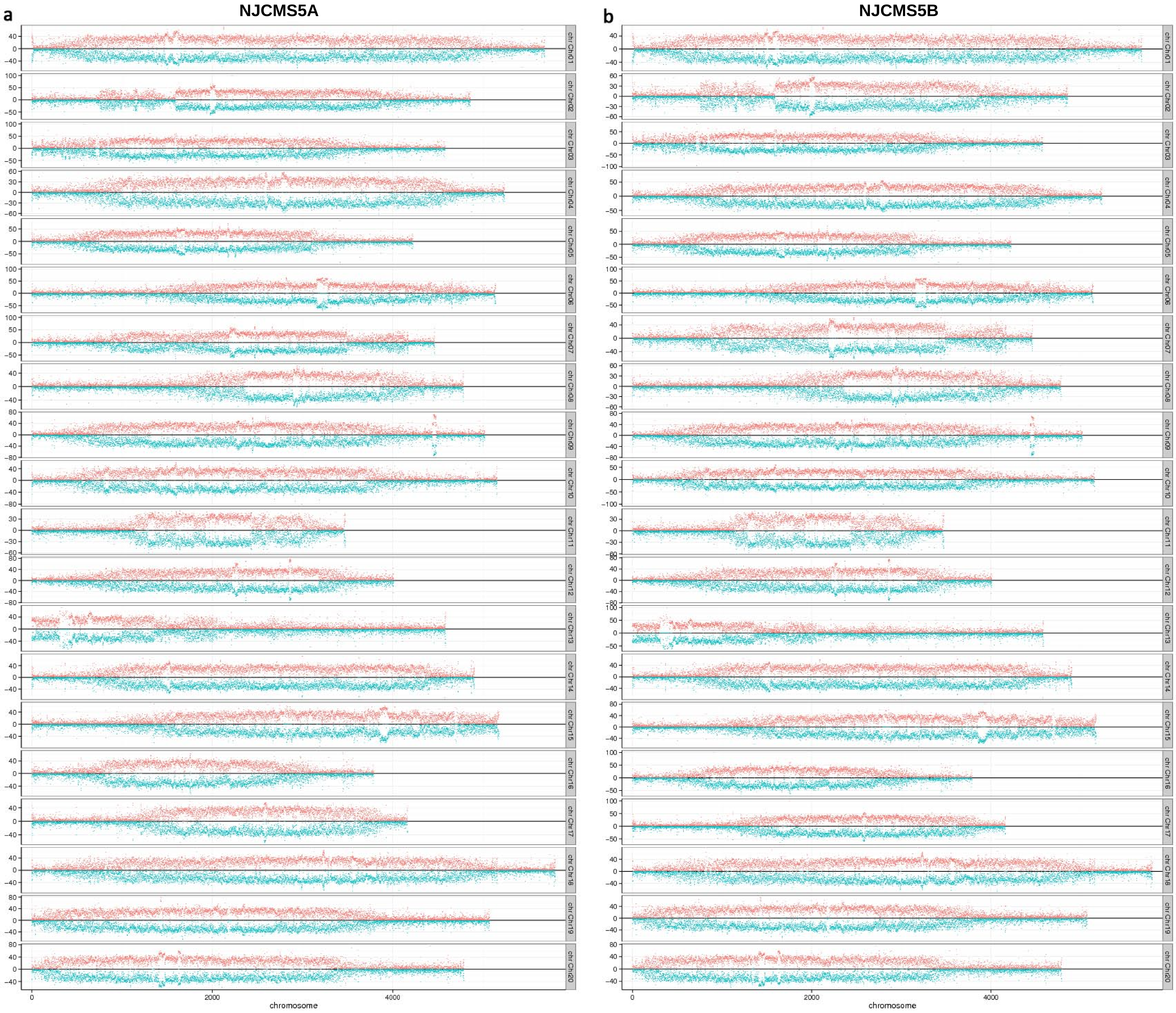

Figure S2 Density distribution of genome-wide methylation in soybean chromosome. (a) NJCMS5A; **(b)** NJCMS5B.

The red indicates positive chain; the green indicates negative chain. Sequencing reads were plotted in 10 kb windows along the chromosome.
