# Supplementary material for: Genome-wide comparative analysis of DNA methylation between soybean cytoplasmic male-sterile line NJCMS5A and its maintainer NJCMS5B
Source: BMC Genomics. 2017 Aug 10;18:596. doi: 10.1186/s12864-017-3962-5 (PMC5557475; doi:10.1186/s12864-017-3962-5)
Supplement: Supplementary file 4 — Graph of gene expression level in gene functional region. (PDF 146 kb) [file 12864_2017_3962_MOESM4_ESM.pdf]

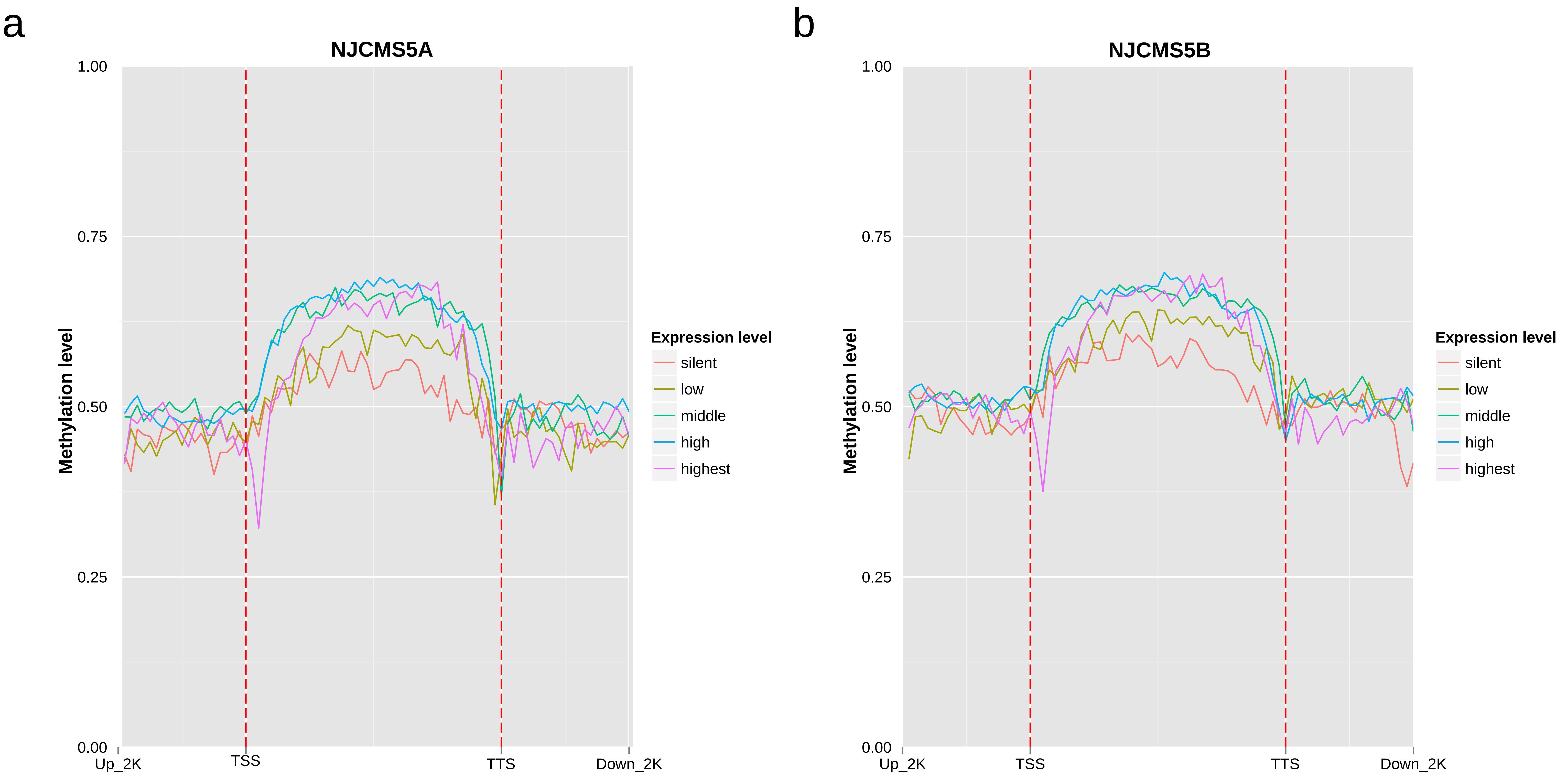

Figure S3 Graph of gene expression level in gene functional region. (a) NJCMS5A; **(b)** NJCMS5B.

Genes was generated from RNA-Seq sequencing and gene expression levels were divided into five groups: silent, low, middle, high and highest. The promoter region defined as the 2 kb region upstream of a transcription start site (TSS) and gene body defined as the region between TSS and TTS.
