# Supplementary material for: Genome-wide comparative analysis of DNA methylation between soybean cytoplasmic male-sterile line NJCMS5A and its maintainer NJCMS5B
Source: BMC Genomics. 2017 Aug 10;18:596. doi: 10.1186/s12864-017-3962-5 (PMC5557475; doi:10.1186/s12864-017-3962-5)
Supplement: Supplementary file 7 — Result of target DMRs in the CG, CHG and CHH context. (DOCX 22 kb) [file 12864_2017_3962_MOESM7_ESM.docx]

**Result of target DMRs in the CG, CHG and CHH context**

| Target region | Sample | Total mC | Sequence context | | |
| --- | --- | --- | --- | --- | --- |
|  |  |  | **mCG** | **mCHG** | **mCHH** |
| Chr01:2321012-2321217 | 5A  5B | 36.645%  25.603% | 94.89%  63.49% | 64.28%  38.88% | 24.13%  17.71% |
| Chr02:218568-218886 | 5A  5B | 24.848%  41.666% | 21.21%  91.66% | 34.09%  68.75% | 24.41%  29.65% |
| Chr03:45134256-451344625 | 5A  5B | 0.468%  0.983% | 1.19%  3.33% | 0%  0% | 0.42%  0.78% |
| Chr04:47863305-47863785 | 5A  5B | 31.282%  37.442% | 80.35%  75.00% | 80.64%  82.24% | 18.42%  26.34% |
| Chr05:6833142...6833444 | 5A  5B | 29.074%  31.842% | 98.61%  98.73% | 70.23%  75.00% | 7.03%  9.34% |
| Chr06:45486978…45487180 | 5A  5B | 15.467%  18.478% | 100%  97.53% | 0%  13.33% | 1.44%  4.34% |
| Chr07:44447645-44448090 | 5A  5B | 0.403%  56.048% | 0%  89.58% | 0%  70.83% | 0.47%  51.41% |
| Chr08:43715049-43715469 | 5A  5B | 10.613%  17.452% | 37.50%  78.57% | 12.50%  32.50% | 4.86%  1.38% |
| Chr09:5361382-5361731 | 5A  5B | 68.965%  38.256% | 66.66%  37.50% | 79.16%  38.92% | 67.01%  38.28% |
| Chr10:47700252-47700644 | 5A  5B | 2.689%  25.078% | 12.50%  97.05% | 5.00%  43.75% | 0.21%  5.70% |
| Chr12:8600124-8600539 | 5A  5B | 4.027%  31.111% | 20.00%  79.16% | 4.16%  31.94% | 0.37%  20.07% |
| Chr13:19743941-19744349 | 5A  5B | 33.611%  1.666% | 85.41%  10.41% | 71.59%  0% | 7.58%  0.44% |
| Chr14:6128619-6128980 | 5A  5B | 61.475%  91.085% | 93.75%  93.33% | 84.37%  100% | 58.56%  90.35% |
| Chr16:8808752-8809095 | 5A  5B | 17.731%  17.320% | 70.47%  70.23% | 26.66%  23.07% | 2.14%  2.95% |
| Chr17:39055838-39056199 | 5A  5B | 6.410%  10.403% | 35.55%  67.14% | 11.11%  25.00% | 3.65%  4.12% |
| Chr19:40543040-40543572 | 5A  5B | 19.863%  32.701% | 49.04%  87.96% | 20.83%  46.66% | 13.51%  18.83% |
| Chr20:1301400-1301819 | 5A  5B | 9.166%  30.208% | 25.00%  96.87% | 18.18%  69.31% | 3.65%  6.70% |
| scaffold_21:694229…694591 | 5A  5B | 16.083%  13.811% | 100%  72.72% | 57.57%  50.00% | 8.68%  7.67% |
| scaffold_22:53285…533225 | 5A  5B | 70.505%  29.154% | 88.63%  90.90% | 87.60%  79.33% | 65.26%  13.02% |
| scaffold_27:331871…332336 | 5A  5B | 20.847%  26.161% | 93.33%  100% | 53.75%  65.00% | 5.33%  7.48% |
| scaffold_97:59397…59659 | 5A  5B | 0.286%  1.432% | 0%  3.12% | 0%  2.94% | 0.35%  1.06% |
