# Supplementary material for: Genome-wide comparative analysis of DNA methylation between soybean cytoplasmic male-sterile line NJCMS5A and its maintainer NJCMS5B
Source: BMC Genomics. 2017 Aug 10;18:596. doi: 10.1186/s12864-017-3962-5 (PMC5557475; doi:10.1186/s12864-017-3962-5)
Supplement: Supplementary file 8 — Validation of sequencing data by bisulfite treatment. (DOCX 16 kb) [file 12864_2017_3962_MOESM8_ESM.docx]

**Validation of sequencing data by bisulfite treatment**

| **Target regions** | **Bisulfite treatment** | | **WGBS** | |
| --- | --- | --- | --- | --- |
|  | **Methylevel**  **ratio** | **Methylevel difference** | **Methylevel**  **ratio** | **Methylevel difference** |
| Chr01: 2321012-2321217 | 1.431 | 0.110 | 2.309 | 0.101 |
| Chr02:218568-218886 | 0.596 | -0.168 | 0.108 | -0.107 |
| Chr03:45134256-451344625 | 0.476 | -0.05 | 0.177 | -0.101 |
| Chr04:47863305-47863785 | 0.835 | -0.062 | 0.217 | -0.107 |
| Chr05:6833142...6833444 | 0.913 | -0.028 | 0.341 | -0.114 |
| **Chr06:42000516…42001028** | **1.274** | **0.006** | **0.431** | **-0.111** |
| Chr06:45486978…45487180 | 0.837 | -0.030 | 0.420 | -0.116 |
| Chr07:44447645-44448090 | 0.007 | -0.556 | 0.021 | -0.104 |
| Chr08:43715049-43715469 | 0.608 | -0.068 | 0.437 | -0.101 |
| Chr09:5361382-5361731 | 1.803 | 0.307 | 3.643 | 0.102 |
| Chr10:47700252-47700644 | 0.107 | -0.224 | 0.499 | -0.133 |
| Chr12:8600124-8600539 | 0.129 | -0.271 | 0.120 | -0.102 |
| Chr13:19743941-19744349 | 20.175 | 0.319 | 8.972 | 0.100 |
| Chr14:6128619-6128980 | 0.675 | -0.296 | 0.350 | -0.106 |
| **Chr15:44965682-44966061** | **1.044** | **0.016** | **0.393** | **-0.105** |
| Chr16:8808752-8809095 | 1.024 | 0.004 | 2.357 | 0.108 |
| Chr17:39055838-39056199 | 0.616 | -0.040 | 0.168 | -0.102 |
| Chr19:40543040-40543572 | 0.607 | -0.128 | 0.409 | -0.104 |
| **Chr19:1093727-1094160** | **1.216** | **0.027** | **0.434** | **-0.111** |
| Chr20:1301400-1301819 | 0.303 | -0.210 | 0.229 | -0.104 |
| scaffold_21:694229…694591 | 1.165 | 0.023 | 3.359 | 0.105 |
| scaffold_22:53285…533225 | 2.418 | 0.414 | 5.363 | 0.127 |
| scaffold_27:331871…332336 | 0.797 | -0.053 | 0.245 | -0.119 |
| **scaffold_530:7334…7609** | **0.988** | **-0.012** | **2.154** | **0.146** |
| scaffold_97:59397…59659 | 0.200 | -0.011 | 0.438 | -0.237 |

Among of them, the results of bisulfite treatment and the whole-genome bisulfite sequencing were not consistent in the regions of Chr06:42000516…42001028, Chr15:44965682-44966061, Chr19:1093727-1094160 and scaffold_530:7334…7609 (blue line).
