# Supplementary material for: Genome-wide comparative analysis of DNA methylation between soybean cytoplasmic male-sterile line NJCMS5A and its maintainer NJCMS5B
Source: BMC Genomics. 2017 Aug 10;18:596. doi: 10.1186/s12864-017-3962-5 (PMC5557475; doi:10.1186/s12864-017-3962-5)
Supplement: Supplementary file 9 — DMGs predicted to be enriched in KEGG pathways. (DOCX 18 kb) [file 12864_2017_3962_MOESM9_ESM.docx]

**DMGs predicted to be enriched in KEGG pathways**

| **Gene ID** | **KEGG ID** | **Stat** | | **Gene Annotation** |
| --- | --- | --- | --- | --- |
| Glycolysis / Gluconeogenesis | | | | |
| Glyma.U036800 | ko00010 | Hypo | groES-like zinc-binding dehydrogenase family protein | |
| Oxidative phosphorylation | | | | |
| Glyma.U021500 | ko00190 | Hypo | vacuolar proton ATPase A1 | |
| Glyma.17G185800 | ko00190 | Hypo | NADH dehydrogenase 5B | |
| Glyma.U010400 | ko00190 | Hypo | pyrophosphorylase 3 | |
| Glyma.19G261100 | ko00190 | Hypo | pyrophosphorylase 1 | |
| Glyma.U041500 | ko00190 | Hypo | NADH dehydrogenase 5B | |
| Photosynthesis | | | | |
| Glyma.15G238800 | ko00195 | Hypo | PSAJ | |
| Glyma.U016600 | ko00195 | Hypo | photosystem I, PsaA/PsaB protein | |
| Glyma.15G238700 | ko00195 | Hypo | photosystem II reaction center protein E | |
| Biosynthesis / Metabolism of amino acids | | | | |
| Glyma.10G248800 | ko00270/ ko00330 | Hypo | s-adenosyl-L-methionine-dependent methyltransferases protein | |
| Glyma.U013100 | ko00290/ko00620/ko01230 | Hypo | 2-isopropylmalate synthase 1 | |
| Glyma.U013200 | ko00290/ko00620/ko01230 | Hypo/Hype | 2-isopropylmalate synthase 1 | |
| Glyma.U016800 | ko00330/ ko00910 | Hypo/Hype | glutamate dehydrogenase 1 | |
| Glyma.14G213200 | ko00330/ko01230/ ko00910 | Hyper | glutamine synthetase 1;4 | |
| Glyma.U031400 | ko00340/ ko01230 | Hypo/Hype | none | |
| Proteolysis | | | | |
| Glyma.U006800 | ko04120/ ko04141 | Hypo | carboxyl terminus of HSC70-interacting protein | |
| Glyma.U008400 | ko04120/ ko04141 | Hypo/Hype | carboxyl terminus of HSC70-interacting protein | |
| Glyma.13G059200 | ko04120/ ko04141 | Hypo | U-box domain-containing protein | |
| Glyma.U012100 | ko04141 | Hypo/Hype | DNAJ homologue 2 | |
| Carbon metabolism | | | | |
| Glyma.02G117500 | ko01200/ ko00620 | Hyper | phosphoenolpyruvate carboxylase 4 | |
| Glyma.02G130700 | ko01200/ ko00620 | Hyper | phosphoenolpyruvate carboxylase 4 | |
| Glyma.19G232600 | ko01200/ ko01230 | Hypo | pyridoxal phosphate (PLP)-dependent transferases protein | |
| RNA activity | | | | |
| Glyma.U010100 | ko03013 | Hypo/Hype | eukaryotic translation initiation factor 2 | |
| Glyma.U016500 | ko03013 | Hypo/Hype | nuclear_porin | |
| Glyma.05G005200 | ko03018 | Hyper | polyribonucleotide nucleotidyltransferase, putative | |
| Glyma.U033400 | ko03018 | Hypo | NOT2 / NOT3 / NOT5 family | |
| Regulation of actin cytoskeleton | | | | |
| Glyma.U034700 | ko04810 | Hypo/Hype | protein kinase superfamily protein | |
| Glyma.U021800 | ko04810 | Hypo | mitogen-activated protein kinase 3 | |
| Circadian rhythm | | | | |
| Glyma.U034500 | ko04712 | Hypo/Hype | mitogen-activated protein kinase 3 | |
| Phenylpropanoid biosynthesis | | | | |
| Glyma.17G052900 | ko00940 | Hypo | peroxidase superfamily protein | |
| Glyma.U017400 | ko00940/ ko00500 | Hypo/Hype | beta glucosidase 42 | |
| Pantothenate and CoA biosynthesis | | | | |
| Glyma.18G223500 | ko00770 | Hypo | flavoprotein | |
| Terpenoid backbone biosynthesis | | | | |
| Glyma.16G118100 | ko00900 | Hyper | undecaprenyl pyrophosphate synthetase family | |
| Carotenoid biosynthesis | | | | |
| Glyma.U016700 | ko00906 | Hypo | carotenoid cleavage dioxygenase 7 | |
| Anthocyanin biosynthesis | | | | |
| Glyma.U001500 | ko00942 | Hypo | UDP-glucosyl transferase 78D2 | |
| Tropane, piperidine and pyridine alkaloid biosynthesis | | | | |
| Glyma.18G186800 | ko00960 | Hypo | NAD(P)-binding Rossmann-fold superfamily protein | |
| Steroid hormone biosynthesis | | | | |
| Glyma.18G011500 | ko00140 | Hypo | beta-ketoacyl reductase 1 | |
| Plant hormone signal transduction | | | | |
| Glyma.16G011800 | ko04075 | Hypo/Hype | SAUR-like auxin-responsive protein family | |
| Porphyrin and chlorophyll metabolism | | | | |
| Glyma.U039000 | ko00860 | Hyper | ferrochelatase 2 | |
| Amino sugar and nucleotide sugar metabolism | | | | |
| Glyma.U025400 | ko00520 | Hypo/Hype | UDP-D-apiose/UDP-D-xylose synthase 1 | |
| Cell cycle | | | | |
| Glyma.13G083100 | ko04110 | Hyper | putative protein kinase 1 | |
| Ribosome | | | | |
| Glyma.U010600 | ko03008 | Hypo/Hype | p-loop containing nucleoside triphosphate hydrolases protein | |
| Glyma.U024000 | ko03010 | Hyper | senescence associated gene 24 | |
| Glyma.U009100 | ko03010 | Hypo | structural constituent of ribosome | |
| RNA polymerase | | | | |
| Glyma.U034000 | ko03020 | Hypo/Hype | nuclear RNA polymerase C1 | |
| Glyma.07G125300 | ko03020 | Hyper | nuclear RNA polymerase C2 | |
| Spliceosome | | | | |
| Glyma.U024300 | ko03040 | Hypo | cyclophilin-like peptidyl-prolyl cis-trans isomerase protein | |
| Glyma.08G108500 | ko03040 | Hypo | G10 family protein | |
| Proteasome | | | | |
| Glyma.14G073000 | ko03050 | Hypo | proteasome alpha subunit F1 | |
| Glyma.18G186900 | ko03050 | Hypo | AAA-type ATPase family protein | |
| Homologous recombination | | | | |
| Glyma.U027200 | ko03440 | Hyper | chromatin remodeling 42 | |
