# Supplementary material for: Genome-wide comparative analysis of DNA methylation between soybean cytoplasmic male-sterile line NJCMS5A and its maintainer NJCMS5B
Source: BMC Genomics. 2017 Aug 10;18:596. doi: 10.1186/s12864-017-3962-5 (PMC5557475; doi:10.1186/s12864-017-3962-5)
Supplement: Supplementary file 10 — Primer pairs used for bisulfite treatment. (DOCX 21 kb) [file 12864_2017_3962_MOESM10_ESM.docx]

**Primer pairs used for bisulfite treatment**

| primers | Primer sequence (5’-3’) | Location | Length | Depth |
| --- | --- | --- | --- | --- |
| Primer1 | F:TGTTATTATTAAGAAGAAAAGAGATTAGGT  R:ACAACAACACCAATCCCTTCATTACAAATTC | Chr01:  2321012-2321217 | 206 | 12.7 |
|  | F:TTAAGAAGAAAAGAGATTAGGTATGATAAT  R:ACAATCCAATCTATTATATCTTATTACACAA |  |  |  |
| Primer2 | F:TTTTAATATAGTTGTTGTTTAGGTTTATTATG  R:TTCAAACTCAATATCTCATCATCATT | Chr02:  218568-218886 | 319 | 14.2 |
|  | F:GTTGTTGTTTAGGTTTATTATGTTATTTAG  R:CTTATCCTATTTATATATATTCTTTTAATTAT |  |  |  |
| Primer3 | F:TGTATATTAGAATGAATAAATTTTCGTTTGATTTG  R:TATTTCTAAAACATATATCTCATATATCACTCTT | Chr03:  45134256-45134625 | 370 | 9.8 |
|  | F:TGATTTGAATTAAAATTAGTTTGGAATGATGG  R:ACTCAATAAATATATACAATTATCTCTATATA |  |  |  |
| Primer4 | F:AATGAATTTATGTTAGTTTAAATATGATAATGG  R:CAATACAACTTAAATTATTTCAATCCAAAATCT | Chr04:  47863305-47863785 | 481 | 11.0 |
|  | F:ATAGAGATATAAGATTATAGTATGAGTTG  R:CCAAATATTTAAACATTTCCTTAAAATCACATC |  |  |  |
| Primer5 | **F:**TGTGGTYATGTGATTTYAATTGGATTGGGT  **R:**CCACATATRCCAATTTCTCCTTCATATCAC | Chr05:  6833142...6833444 | 302 | 13.4 |
|  | **F:**TAAATGTTATATGTTGGYTGAGTT  **R:**CTCTCAATAAAAATTTTCACCAAATACT |  |  |  |
| Primer6 | **F:**AAATATGGTAAAGAAYATGGGGATAAAGATGAGT  **R:**AATCAAATCTACATCCATCCARATCCTC | Chr06:  42000516…42001028 | 512 | 8.6 |
|  | **F:**TGGTTAAATGGGAAAATTTTTTGTA  **R:**CTTAATTCATTTCTCTRATTAACCATATTATTAC |  |  |  |
| Primer7 | **F:**TTAGGTGGTTTGGGYATGTAGAGAGAAGA  **R:**ACTTACCTCCCAAATCAACCCAAATTCT | Chr06:  45486978…45487180 | 202 | 10.1 |
|  | **F:**AGGTAGAGGAAGATTTAAAAAGATTATAAG  **R:**TCACAAACATATTTTCCCCAATATTA |  |  |  |
| Primer8 | F:TAATAAATATTTGATTAAAGGTGTAAATATTG  R:CACTTCAACAAAATCTCCACAATAACTTTTAA | Chr07  44447645-44448090 | 446 | 14.1 |
|  | F:AGGTGTAAATATTGAATTATTTTTTTTTGAAG  R:AATTTTTTAACTAAAATAATTTTTCCATCTAA |  |  |  |
| Primer9 | F:TTTTATTTTATATGTAGAAAAAATGTGAAGTAG  R:TCATTCTCCATTTATTTTCCTATACACTCACAT | Chr08:  43715049-43715469 | 421 | 13.2 |
|  | F:AAGTAGTAATTAGATATTGATGATAAATAG  R:CTTAATTTCCATCTCCTCACCCTAAA |  |  |  |
| Primer10 | F:AGTAGTATTGTAGAGGAGGGGTTTTG  R:TCTCTTACTCTTAATTAACTATCATATTCT | Chr09:  5361382-5361731 | 350 | 11.0 |
|  | F:ATGGTTGGTGGAATTGATGGAGAAAT  R:CACTTTTAATCCCCTACTTTAACTCTAATTTT |  |  |  |
| Primer11 | F:GAAAGGAGAAGAAGAGTATGAGAGAGAA  R:AATTATACAACAACCAATTCAATTCTACA | Chr10:  47700252-47700644 | 393 | 10.5 |
|  | F:GGAAATGTGTAATGTGTATAAAGAAGGAAG  R:CAATTCAATTCTACAACAACAATTATTTAC |  |  |  |
| Primer12 | F:ATGAGATAAATAATTTTGTAAAATATTTGATAA  R:CATTTCCATCCAATTAATTTACATAATAATAAT | Chr12:  8600124-8600539 | 416 | 9.3 |
|  | F:ATTTGATAATTAATTTTGGGAAATATTTGGTAA  R:CAATCAATCCTAACACCAAATCCAAAATCAACA |  |  |  |
| Primer13 | F:GATGTTGAAAGTATGAAAATTGAAGAATTGTG  R:TCACAATATTTCCCATACCTTCTACT | Chr13:  19743941-19744349 | 408 | 11.2 |
|  | F:TTAAGAAAGGTAGTAGTGATAAAAAGAAAT  R:TATCAAAATCAATTAACCAATCTTTAC |  |  |  |
| Primer14 | F:TAATAGAGATATTTTTGAATGATATTGAA  R:TTAATATTCATACTCCATAATCCCTAATT | Chr14:  6128619-6128980 | 362 | 12.3 |
|  | F:TTGAATGATATTGAATTAAAAGATGATAA  R:ATAATTCTTTCTACCTCCATTACATCAC |  |  |  |
| Primer15 | F:TATAATTTAAATTAGGTTTATTGTTGGATGAA  R:ATTACATTTCAATAAATTTCTACTTACT | Chr15:  44965682-44966061 | 380 | 10.9 |
|  | F:ATTATATTGTAAAAATTGGATAGGT  R:TAAATTTCTACTTACTAATCTCATCTTTTAT |  |  |  |
| Primer16 | F:TGATGGGTAAGTTGTTGTGTTTGATGA  R:CTTTCCTACATTTTCAATTACTTCAAATCA | Chr16:  8808752-8809095 | 344 | 9.4 |
|  | F:TTTTTTGTTGATAAAGTGTAGGAGA  R:AACCAAAATCTACTCATTATCATTACCT |  |  |  |
| Primer17 | F:GGATGGAATGTAATATGGTTGGTGG  R:CCAAATAAATACAACAATCATTACAAATTC | Chr17:  39055838-39056199 | 362 | 10.0 |
|  | F:AAGTGTTAATAAGTTTTGATATTGGT  R:TCATTACAAATTCCATTTCTAATTC |  |  |  |
| Primer18 | F:TTTGATTAGTAGAGGAAAAAGTAAGTGTG  R:CCCTATATTATTTTCTAAATTAATTAATACTTA | Chr19:  40543040-40543572 | 533 | 10.8 |
|  | F:AAAGATGAGAAATAAAAAAGTTGTGTGAGATTT  R:AAAAATTCTATTCTTACTTAATTCC |  |  |  |
| Primer19 | F:TGGAATAAAAAATATTATTGGGGAAAGGATAT  R:CTCAATCATATTATATATATATTAATTT | Chr19:  1093727-1094160 | 434 | 11.7 |
|  | F:TGTAATTGTTATTATATAAATGTTTTAGAG  R:CAATAAATTAAAATAACCAATACCTTCTTCT |  |  |  |
| Primer20 | F:AAGGGTGAGATAATGTGGGAGATAAAGG  R:CTCCTTCAATATAAATAATTTCACTACAATCTT | Chr20:  1301400-1301819 | 420 | 11.7 |
|  | F:GAGATAAAGGTTGAATGTTAAATTGG  R:CATATTAAATCATTTTTAACATTCACAAACTAA |  |  |  |
| Primer21 | **F:**AGGTTTAGGGGAGTTTYTGAATTGG  **R:**TTAACATCTTCCAATCTTCACATC | scaffold_21:  694229…694591 | 362 | 10.5 |
|  | **F:**AAAGYAAGAGAATGTTGTGTYAGGAGAA  **R:**CCCATCCCCATCACCATCATCRATRTTRTCATT |  |  |  |
| Primer22 | **F:**AGAGAAAATATTTGAAAAATGATTGTGAGAAGTT  **R:**AAAATTTTATAAAAAACTTATCACAACCCA | scaffold_22:  532779…533245 | 466 | 11.7 |
|  | **F:**TAGGGTGTGATTTTTGGTTTTAGAAA  **R:**TATAAAAAACTTATCACAACCCARTTTCCTATCA |  |  |  |
| Primer23 | **F:**TAATGTGTAYGAATGGGGGATAAGTGAATAT  **R:**CATCTTTCTCTRCACTRTTTACCAATCCTACC | scaffold_27:  331871…332336 | 465 | 11.4 |
|  | **F:**AGGAYATTGATTGGAAGGTAAAGATGA  **R:**TTTACCAATCCTACCATRCAATAACTTT |  |  |  |
| Primer24 | **F:**AAGAATGATGATGATGATGAGGTTTGGAAGAT  **R:**CCAATTTCATCTCCTTTCCTTAAACCCCTTCT | scaffold_530:  7334…7609 | 275 | 8.8 |
|  | **F:**TGATGAGGTTTGGAAGATYAAGAAAGTGTTGG  **R:**CCTTTCCTTAAACCCCTTCTTRCAACAAAATC |  |  |  |
| Primer25 | **F:**TTGTTGATTGTGAYAYAAAGGGTGTGGA  **R:**TTCCCCATRARTTCTTAACCAACCAA | scaffold_97:  59397…59659 | 262 | 12.4 |
|  | **F:**TGTGATATAAAGGGTGTGGATTAAG  **R:**TCAAAACCACTAACATCAATAACTAC |  |  |  |
