# Supplementary material for: Genome-wide comparative analysis of DNA methylation between soybean cytoplasmic male-sterile line NJCMS5A and its maintainer NJCMS5B
Source: BMC Genomics. 2017 Aug 10;18:596. doi: 10.1186/s12864-017-3962-5 (PMC5557475; doi:10.1186/s12864-017-3962-5)
Supplement: Supplementary file 11 — Primer pairs used for quantitative real-time PCR (qRT-PCR). (DOCX 17 kb) [file 12864_2017_3962_MOESM11_ESM.docx]

**Primer pairs used for quantitative real-time PCR (qRT-PCR)**

| primers | Gene name | Primer sequence (5’-3’) | Length |
| --- | --- | --- | --- |
| Primer1 | β-Tublin | F: GGAGTTCACAGAGGCAGAG  R: CACTTACGCATCACATAGCA | 189 |
| Primer2 | Glyma.06G248800 | F: ACCCTTGTTCTAATGGCCAGTG  R: TCAGAGCAAGATAAGCCACAAGT | 144 |
| Primer3 | Glyma.U045200 | F: ACAAAGGGTGTGGACCAAGG  R: TTGTTGGCAGGGACATCCTC | 188 |
| Primer4 | Glyma.06G266900 | F: CTCAAGTGGCATACCGAAACAG  R: ACCAAGGCAGGGACAAGTTCT | 117 |
| Primer5 | Glyma.08G305500 | F: AGGTAAACCCAAGAAAGCGAT  R: CTCCAAATCCAAGCCATTACCT | 146 |
| Primer6 | Glyma.16G195100 | F:TGGGCTGTGCAAGAGTGG  R: AGCGCTTCATCAAACAAGCC | 139 |
| Primer7 | Glyma.14G212600 | F: CCTGCACTCACCTTCTTCAATG  R: TTCTCGGAATCGTTGATGGCT | 100 |
| Primer8 | Glyma.U029400 | F: GGTGGCATAGGAAAGGATAAGTT  R: CACCACCAACTATACAACACAAG | 125 |
| Primer9 | Glyma.U040000 | F: GGAGTTTGTGTTGCCTTGGAG  R: TCTCCTTTCCTTAAACCCCTTC | 159 |
| Primer10 | Glyma.U013000 | F: TGGAGGAGAAACCATGACTGAA  R: TCTTCACATCAATCCCATCCC | 122 |

β-Tublin as the internal control gene
